# Fight against tobacco in Japan, before and after FCTC

**DOI:** 10.1186/1617-9625-12-S1-A29

**Published:** 2014-06-06

**Authors:** Kazunari Satomura

**Affiliations:** 1 Department of Public Health, Faculty of Medicine, Kyoto University, Kyoto, 606-8501 Japan

## Background

In 1965 smoking rate of male in Japan was 82.3% and that of female was 15.7%. In 2005 when the FCTC was enacted, smoking rate of male was 39.3% and that of female was 11.3%. In 2013 smoking rate of male is 32.2% and that of female is 10.5%. It seems that several measures against smoking for the FCTC accelerate to reduce the smoking rates. To find effective measures for decrease smoking prevalence, anti-tobacco measures before and after the FCTC are checked.

## Results

### (Before the FCTC)

From 1898 to 1985, tobacco was under monopoly. In that period, Act of Prohibiting Minors from Smoking is a law worthy of mention. It was enacted in 1900 and revised several times, but even now smoking of minors is illegal. This law has some effect on reducing minors’ smoking, but minors who want to act like adults become to smoke. Since ‘60s negative health effects of smoking become to be known. In 1967, volumes of tar and nicotine in cigarettes are printed on the package. Also warning: “Please be careful about smoking too much “becomes to be printed on the package in 1972. Since ‘70s, anti-smoking activities become popular. Some smokers become aware of negative health effects of smoking and changed their cigarettes named “light” or “mild”. The policies at that period were ”reducing smoking”, “separating smoking place” and “stopping smoking”. Diffusing negative health effect of smoking reduces smoking prevalence gradually.

### (After the FCTC)

TASPO which is a kind of ID card was introduced to prevent minors from buying tobacco by vending machine in 2008. Number of tobacco vending machines was decreased by it. Warning messages become to be printed on the packages. They are scientific but give small impact for smokers. The prices of cigarettes are raised. The policies are changed to “separating smoking place”, “stopping smoking”,” preventing minors from becoming smokers”. Smoking is recognized as a disease and the medical fee for quitting smoking are paid by the medical insurance.

## Conclusions

These measures are insufficient. A large proportion of people wants to become healthy and hates passive smoking. This trend makes to reduce smoking places and smokers.

